# Variations in medical practice of retinopathy of prematurity among 8 Asian countries from an international survey

**DOI:** 10.1038/s41598-023-42432-3

**Published:** 2023-09-20

**Authors:** Young-Ah Youn, Sae Yun Kim, Su Jin Cho, Yun Sil Chang, Fuyu Miyake, Satoshi Kusuda, Adhi Teguh Perma Iskandar, Rinawati Rohsiswatmo, Rizalya Dewi, Seok Chiong Chee, Siew Hong Neoh, Ma. Lourdes S. Imperial, Belen Amparo E. Velasco, Bin Huey Quek, Yuh-Jyh Lin, Jui-Hsing Chang, Pracha Nuntnarumit, Sopapan Ngerncham, Sarayut Supapannachart, Yuri Ozawa, Seiichi Tomotaki, Chatchay Prempunpong, Pathaporn Prempraphan, Tetsuya Isayama

**Affiliations:** 1 Department of Pediatrics, College of Medicine, The Catholic University of Korea, Seoul, Republic of Korea; 2 Department of Pediatrics, Ewha Womans University College of Medicine, 1071 AnYang Cheon-ro, YangCheon-gu, Seoul, 07985 Republic of Korea; 3 Department of Pediatrics, Samsung Medical Center, Sungkyunkwan University School of Medicine, 81 Irwon-ro, Gangnam-gu, Seoul, 06351 Republic of Korea; 4 Division of Neonatology, National Center for Child Health and Development, Tokyo, Japan; 5 Department of Pediatrics, Neonatal Research Network of Japan, Kyorin University, Tokyo, Japan; 6 Department of Child Health, Faculty of Medicine Universitas Indonesia, Cipto Mangunkusumo General Hospital, Jakarta, Indonesia; 7 Budhi Mulia Mother and Child Hospital, Pekanbaru, Indonesia; 8 Department of Pediatrics, Selayang Hospital, Ministry of Health, Batu Caves, Kuala Lumpur, Malaysia; 9 Department of Paediatrics, Hospital Tunku Azizah, Ministry of Health, Kuala Lumpur, Malaysia; 10 Dr. Jose Fabella Memorial Hospital, Manila, Philippines; 11 Philippine Children’s Medical Center, Manila, Philippines; 12 Department of Neonatology, KK Women’s and Children’s Hospital, Singapore City, Singapore; 13 Department of Pediatrics, National Cheng-Kung University Hospital, Tainan, Taiwan; 14 Department of Pediatrics, MacKay Memorial Hospital, Taipei, Taiwan; 15 Department of Pediatrics, Faculty of Medicine Ramathibodi Hospital, Mahidol University, Bangkok, Thailand; 16 Department of Pediatrics, Faculty of Medicine Siriraj Hospital, Mahidol University, Bangkok, Thailand; 17 Division of Neonatology, National Center for Child Health and Development, Department of Pediatrics, Kyorin University, Tokyo, Japan; 18 Department of Pediatrics, Graduate School of Medicine, Kyoto University, Kyoto, Japan; 19 Department of Pediatrics, Faculty of Medicine Vajira Hospital, Navamindradhiraj University, Bangkok, Thailand

**Keywords:** Health care, Medical research

## Abstract

Advances in perinatal care have led to the increased survival of preterm infants with subsequent neonatal morbidities, such as retinopathy of prematurity (ROP). This study aims to compare the differences of neonatal healthcare systems, resources, and clinical practice concerning ROP in Asia with review of current literature. An on-line survey at the institutional level was sent to the directors of 336 neonatal intensive care units (NICU) in 8 collaborating national neonatal networks through the Asian Neonatal Network Collaboration (AsianNeo). ROP screening was performed in infants born at < 34 weeks in Indonesia and Japan. In South Korea, Malaysia, and Taiwan, most screened for ROP in infants born at < 32 weeks. In all networks, majority of NICUs conducted ROP screening to infants with birth weight < 1500 g. In most NICU’s in-hospital ophthalmologists performed indirect ophthalmoscopy and some were supplemented with digital imaging. Both laser photocoagulation and anti-vascular endothelial growth factor injection are performed for treatment and, vitreous surgeries are conducted less frequently in all countries. Despite limited information collected by the survey, this first study to compare ROP practices implemented in eight Asian countries through AsianNeo will enable an understanding of the differences and facilitate quality improvement by sharing better practices.

## Introduction

Retinopathy of prematurity (ROP) was firstly described as a syndrome, retrolental fibroplasia in eight preterm infants by Terry^1^. It is a disease characterized by irreversible visual consequences due to abnormal retinal vascularization, potentially leading to retinal detachment and severe vision impairment or blindness if left untreated^2^. The World Health Organization reported in 2022 that approximately 15 million neonates are born prematurely each year^3^. Over the past 2 decades, the mortality rate among very or extremely preterm infants has decreased due to enhanced neonatal care. However, the surviving infants still face a lifelong risk of disabilities, including blindness. Each year, around 32,300 infants worldwide are diagnosed with irreversible vision impairment caused by ROP, of which about 20,000 become blind or severely visually impaired^2^. Given the inability to completely prevent premature birth, ROP ranks among the leading causes of childhood blindness. Hence, early detection of severe ROP and timely intervention are crucial. While the foundation for reducing in ROP-related blindness lies in appropriate, universally implemented ROP screening, progress in ROP screening programs has not matched advancement in neonatal care. Currently, established ROP screening protocols and the availability of trained ROP specialists for preterm infants vary significantly between countries, particularly between high-income and low-to-middle income countries.

The history of ROP comprises three distinct epidemics. The first epidemic was from the late 1940s to early 1950s, caused by high fraction of inspired oxygen without adequate monitoring to the preterm infants^4^. The second epidemic emerged in the late 1960s and early 1970s, in high-income countries. Improved neonatal care increased the survival chances of preterm infants with lower GA, resulting in ROP cases despite efforts at titration of oxygen concentration^5,6^. In the early 1990s, a non-uniform pattern of the disease was detected, called the third epidemic. Developed countries saw ROP risks primarily in extremely preterm infants, while middle-income countries with evolving neonatal intensive care witnessed a rise in ROP-related blindness^7^. Our survey across Asian countries underscores the substantial burden of ROP, coupled with diverse of screening criteria.

Neonatal networks play a crucial role in elevating care standards by providing evidence-based feedback and establishing national healthcare policies based on local population data. These networks also foster collaboration among neonatal researchers, aiding the implementation of evidence-based strategies to reduce mortality and morbidity in high-risk infants^8^. The neonatal networks are able to leverage on evidence-based strategies to decrease mortality and morbidities of high-risk infants^9^. Globally, several renowned national and international neonatal networks are active, including the National Institute of Child Health and Human Development (NICHD) Neonatal Research Network (NRN), Vermont Oxford Neonatal Network (VON), Australian and New Zealand Neonatal Network, Canadian Neonatal Network, Neonatal Research Network Japan (NRNJ), EuroNeoNet, and Korean Neonatal Network (KNN)^8^. These networks facilitate extensive population-based data collection and healthcare statistics at national or international levels. Despite notable improvements in outcomes, significant variation persists both within and between networks. Collaborative sharing of information across diverse neonatal network from different countries and regions can estimate practice diversity, offer evidence for practices, and monitor practice implementation, thereby potentially enhancing neonatal outcomes and reducing global healthcare costs^10^.

Notably, the International Network for Evaluation of Outcomes (iNeo) in neonates was established to promote a quality improvement project through collaborative international comparison of population-based neonatal health services, specifically for extremely preterm/extremely low birth weight neonates^10^.

Premature births are not equally distributed across the world, with more than half of the countries with the highest number of premature births located in Asia^11^. The Asian region encompasses both high-income countries like Japan and South Korea and low-income countries such as Nepal and Cambodia, with many others falling within the middle-income category, including the populous countries of China and India. Given this heterogeneous mix of characteristics, comparative research through international collaboration within neonatal network is imperative. This study aims to provide an overview of ROP-related clinical protocols, neonatal intensive care unit (NICU) facilities, human resources, screening policies, and treatment strategies across eight Asian countries. The data was collected through an online survey conducted by AsianNeo, aiming to shed light on differences in perinatal and neonatal healthcare systems, compare resources for ROP management, and analyze subsequent outcomes among these Asian countries. The secondary objective involves describing recent epidemiological trends, such as incidence rates and screening policies, specific to each country.

## Results

### A recent survey of AsianNeo about ROP related clinical practices

There were four national neonatal networks registry of very low birth weight (VLBW) infants and four national population based neonatal network in the eight countries participating in the current survey study by the AsianNeo: the network from Indonesian pediatric society, NRNJ, KNN, Malaysian National Neonatal Registry (MNNR), the network from Philippine Society of Newborn Medicine, the network from level III to IV public hospitals in Singapore, Taiwan Neonatal Network (TNN), and the network from Thai Neonatal Society. Eight Asian countries had similarities and differences in neonatal intensive care. The lowest gestational age (GA) for neonatal resuscitation in eight Asian countries varied from 22 to 26 weeks, Infants of South Korea and Japan were provided resuscitation from the youngest, born at 22 weeks of gestation, and infants of Indonesia were provided resuscitation from the oldest, born at 25–26 weeks or older. The lowest birth weight of infants resuscitated were also varied between 250 and 600 g.

Neonatal resuscitation guidelines were based on the AAP’s neonatal resuscitation guideline in all 8 countries. Five countries made modifications to fit local practices. More than half of the units participated in the survey were university hospitals: twenty-eight for Indonesia, sixty-two in Japan, twelve in South Korea, one for Malaysia, seven for Philippines, three for Singapore, fifteen in Taiwan, and thirty-three for Thailand. The level of NICU was mostly more than II. The most common upper Saturation of Peripheral Oxygen (SpO_2_) target limit was 95% (range 94–97%) and lower SpO_2_ target was 90% (range 88–91%) in the responded NICUs (Table 1).

**Table 1:** Baseline Information of neonatal care and Facilities and ROP related strategies per country comparison with US/UK.

Country	Baseline information of participating networks and countries						Information on units(hospital) participating in the survey					
	NRP	Resuscitated ≥ GA or BW		Neonatal network registry of VLBW infants*		Reported ROP incidences	UH	Level			SpO_2_ limit, median (IQR)	
		GA, wk	BW, g	Existence	Inclusion criteria, (GA,wk/BW,g)			III	II + III	Others	Lower	Upper
Indonesia	Indonesian NRP	25–26	600	NO		Any ROP, 6.7% (228/3425) GA < 34^+0^ wks and/or BW < 1500 g^12^	28/38	6	30	2	90 (88, 90)	95 (94, 95)
Japan	Japanese NRP	22	250–300	NRNJ	GA < 32 or BW < 1500	Treated ROP, 14.8% (5268/35,536), BW < 1500 g, NRNJ database^13^	62/144	61	74	10	88 (86, 90)	95 (95, 97)
South Korea	Korean NRP	22		KNN	GA < 32 or BW < 1500	Treated ROP 11.5% (231/2009), BW < 1500 g, KNN database^14^	12/13	4	8	1	89 (88, 90)	95 (95, 96)
Malaysia	AAP's NRP	24	500	MNNR	GA < 32 or BW < 1500	Any ROP 13.0% (261/2007), GA < 32 wks, LOS > 6 week, MNNR 2018 database	1/35	4	28	3	90 (89, 91)	95 (94, 95)
						Any ROP 29.4% (94/320) in 2010–2016, GA ≤ 32 + 0 wk or BW < 1501 g, single tertiary center ^15^						
Philippines	NRPhPlus	24–25	400	NO	–	Any ROP 13.9% (118/851) GA < 35 wk, single tertiary center^16^	7/16	2	11	3	90 (89, 90)	94 (94, 95)
Singapore	Singapore NRP	23	400	NO^†^	–	Severe ROP, 8.6% (21/244, 2007) and 8.9% (62/699, 2017), BW < 1500 g, national cohort study^17^	3/3		3		89 (89, 90)	94 (94, 95)
Taiwan	AAP's NRP (modified)	22–23	300–400	TNN	GA ≤ 29 or BW 401–1500	Any ROP 36.6% (4096/11,180), GA < 37^+0^wk, LOS > 28 d, data from the NHIRD^18^	15/25	8	13	4	88 (88, 90)	95 (95, 95)
Thailand	AAP's NRP	23	500	NO	–	Any ROP 17.7% (20/113)(GA ≤ 30 wks or BW ≤ 1500 g), single tertiary center^19^	33/62	11	49	2	90 (90, 90)	95 (95, 95)
US	AAP's NRP	24		NICHD NRN	BW < 1500	Any ROP 70.6% (2413/3416), treated ROP 13.7% (469/3416), GA < 27^+0^wk,NICHD registry^20^						
						16.4%, LOS > 28d, GA, BW not specified, nationwide database^21^						
UK	Resuscitation Council UK	24		NNRD	All GA	treated ROP 4.0% (327/8112), BW < 1500 g, BOSU database^22^						
						Any ROP 12.6% in 2011 BW < 1500 g, a dataset from the NHS^23^						

*AAP* American Academy of Pediatrics, *BOSU* British Ophthalmic Surveillance Unit, *BW* birth weight, *GA* gestational age, *KNN* Korean neonatal network, *LOS* length of stay, *MNNR* Malaysian National Neonatal Registry, *NHS* National Health Service, *NICHD* National Institute of Child Health and Human Development, *NHIRD* The National Health Insurance Research Database in Taiwan, *NNRD* National Neonatal Research Database, *NRNJ* Neonatal Research Network of Japan, *NRP* neonatal resuscitation program, *NRPhPlus* NRPh+, Principles of Neonatal Resuscitations in Philippines, *ROP* retinopathy of prematurity, *SpO*_*2*_ saturation of peripheral oxygen, *TNN* The Taiwan Neonatal Network, *UH* university hospital, *UK* United Kingdom, *US* United States, *VLBW* very low birth weight, *wk* week.

^†^There is no national registry of VLBW infants in Singapore. However, only 3 NICUs manage more than 80% of VLBW infants born in the country and each of them have their own unit database of VLBW infants.

### Reported incidences of ROP

The incidence of ROP varied by country and by the demographics of the study population (Table 1). In US, a study using the NICHD NRN database, in infants under 27 weeks of gestation, 70.6% (2413/3416) were diagnosed with any stage of ROP^20^. Among newborns admitted in NICU more than 28 days, the incidence was 16.4% in the studies using the publicly available nationwide databases^21^. There were two nationwide studies in the UK, among VLBW infants with a GA < 32 weeks, the incidence of ROP in 2011 was 12.6%^23^ and another study investigated the incidence was 4.0% of treated ROP among VLBW infants^22^. In South Korea, Hwang et al. reported that the incidence of treated ROP in VLBW infants registered in KNN database between 2013 and 2014 was 11.5%^14^. According to the study by Kono et al. the incidence of treated ROP was 14.8% among NRNJ registered VLBW infants between 2003 and 2012^13^. A recent study in Taiwan reported the incidence of any stage ROP 36.6% among premature infants with LOS of more than 28 days using the Nationwide data^18^.

### Published ROP screening guidelines

In US, all infants with a birth weight of ≤ 1500 g or a GA of ≤ 30^+6^ weeks and selected infants with a birth weight between 1500 and 2000 g or a GA of > 30^+6^ weeks who are believed by their attending pediatrician or neonatologist to be at risk for ROP should be screened for ROP^24^. The more premature an infant is at birth, the longer the time to develop severe ROP. Therefore, the onset of severe ROP correlates better with postmenstrual age (PMA) than with postnatal age. Hence, the initiation of ROP screening is recommended to be at PMA 31 weeks for infants born under 27^+6^ weeks GA. Infants with older GA should be initially screened 4 weeks after birth. The UK guideline on ROP has been updated, recently, like the US. All infants with a birth weight of < 1501 g or a GA of < 31^+0^ weeks should be examined to screen for the presence of ROP^25^. For infants born < 31^+0^ weeks’ GA, the first examination is performed between 31^+0^ and 31^+6^ weeks of PMA, or at postnatal 4 completed weeks (28–34 days), whichever is later. For infants born at and after 31^+0^ weeks’ GA with birthweight less than 1501 g, the first ROP examination should be performed at 36 weeks’ PMA or 4 completed weeks’ postnatal age (28–34 days), whichever is earlier. When Asian countries were compared to US and UK, the published screening criteria for GA were < 34 weeks’ GA in Indonesia^26^, ≤ 32 weeks’ GA in Philippines^27^ and Singapore^28^, < 32 weeks’ GA in Malaysia^29^ and Taiwan^30^, < 31 weeks’ of gestation in Japan^31^, and < 30 weeks’ of gestation in South Korea^32^ and Thailand^19^. Majority of NICUs conducted ROP screening to infants with birth weight < 1500 g.

In US and UK, both digital photographic retinal images and binocular indirect ophthalmoscopy (BIO) can be used for screening. However, in both US and UK it is recommended that indirect ophthalmoscopy be performed at least once by an experienced ophthalmologist before treatment or termination of acute-phase screening or ROP for infants at risk for ROP: in UK, final screening examination should be performed using BIO (Table 2).

**Table 2:** Published ROP screening guidelines from eight AsianNeo countries compared with US and UK.

Country	Study	Screening criteria, GA (week) and BW (g)	Timing of first exam	Method	Approval body
Indonesia	Siswanto et al.^26^	GA ≤ 34 and/or ≤ 1500 g GA > 34 with unstable clinical course	GA > 30 wks: PNA 2–4 weeks GA ≤ 30 wks: PNA 4 week	BIO by ophthalmologist	National Neonatologist-ophthalmologist workshop
Japan	Ikeda et al.^31^	GA < 31 and BW < 1500	PNA 3–7 wks	BIO by ophthalmologist	Center guideline
South Korea	Choi et al.^32^	GA ≤ 30^+0^ and/or BW < 1500 BW > 1,500 with unstable course	PNA 4 wks or PMA 31–32 wks, (earlier, minimum PMA 28 wks)	BIO by ophthalmologist	Center guideline
Malaysia	Ministry of Health Malaysia et al.^29^	GA < 32 or < 1500 GA ≥ 32 with unstable clinical course	PNA 4–6 wks	BIO by ophthalmologist	Governmental institution
Philippines	Strategy PPCP et al.^27^	GA ≤ 32 or ≤ 1500 Mature/larger infant with unstable clinical course	GA < 28 wks: at PMA 31 wks GA ≥ 28, PNA 20 days	BIO by ophthalmologist	PPS, PSNbM, PAO-ROPWG
Singapore	Shah et al.^28^	GA ≤ 32, BW ≤ 1500 Mature/larger infant with unstable clinical course	PNA 6 wks of age or PMA 34 wks (earlier)	BIO by ophthalmologist	Center guideline
Taiwan	Chen et al.^30^	GA < 32 or BW < 1500 Mature/larger infant with unstable clinical course	PNA 4–6 wks after birth	BIO, ophthalmologist	Center guideline
Thailand	Mantapond Ittarat et al.^19^	GA ≤ 30, or BW ≤ 1500 Mature/larger infant with unstable clinical course	PNA 4–6 wks or PMA 31–33 weeks	BIO, ophthalmologist	Center guideline
US	Fierson et al.^24^	GA ≤ 30 and/or ≤ 1500 GA > 30 or BW 1500–2000: if unstable clinical course	GA < 27 wks: at PMA 31 wks GA ≥ 27 wks: PNA 4 wks	BIO by ophthalmologist	AAP, American Academy of Ophthalmology
UK	Wilkinson et al.^25^	GA < 31^+0^ and/or < 1501	GA < 31^+0^ wks: PMA 31 wks or PNA 28–34 days (later) GA ≥ 31^+0^ wks: PMA 36 wks or PNA 28–34 days (earlier)	BIO by ophthalmologist or WFDRI	RCPCH, RCOphth

*AAP* American Academy of Pediatrics, *BIO* binocular indirect ophthalmoscopy, *BW* birth weight, *GA* gestational age, *PAO-ROPWG* Philippine Academy of Ophthalmology ROP working group, *PMA* postmenstrual age, *PNA* postnatal age, *PPS* Philippine Pediatric Society, *PSNbM* Philippine Society of Newborn Medicine, *RCPCH* Royal College of Paediatrics and Child health, *RCOphth* Royal College of Ophthalmologists, *ROP* retinopatny of prematurity, *UK* United Kingdom, *US* United States, *WFDRI* wide-field digital retinal imaging, *wk* week.

Most countries like in Asia, Japan^31^, South Korea^32^, Malaysia^29^, Phillipines^27^, Singapore^28^, Taiwan^30^, and Thailand^19^ follow screening guidelines similar to the UK/US: all infants with GA of 30–32 weeks or less and/or with a birth weight of ≤ 1500 g or less should be screened for ROP. However, in Indonesia, infants with higher GA, < 34 weeks of gestation, are screened^26^.

### ROP screening policies in Asian countries

**Figure 1:**
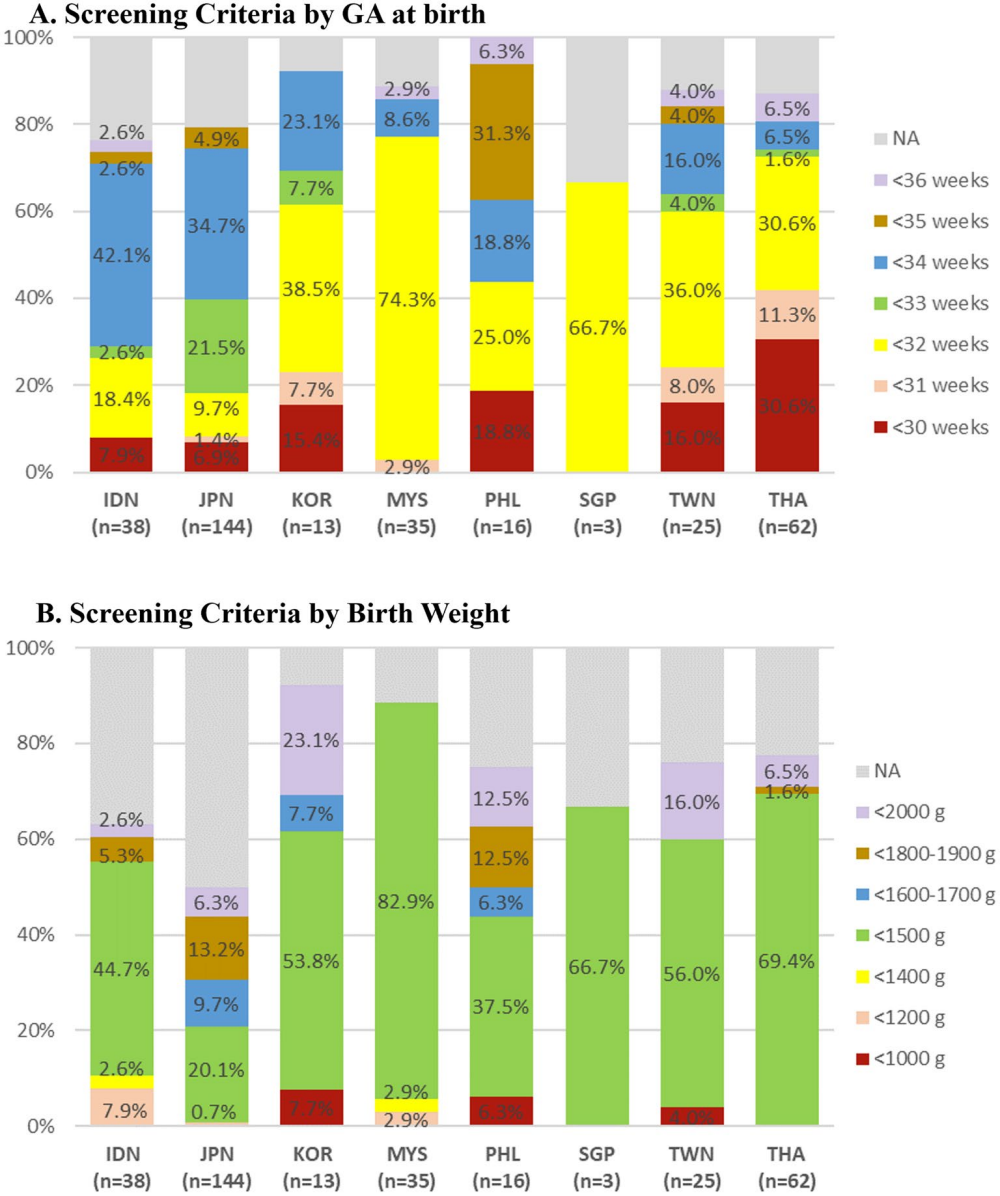
ROP screening criteria in the eight AsianNeo Countries. (**A**) The screening criteria of gestational age for ROP. (**B**) The screening criteria of birth weight. *GA* gestational age, *IDN* Indonesia, *JPN* Japan, *KOR* South Korea, *MYS* Malaysia, *NA* not available, *PHL* Philippines, *SGP* Singapore, *TWN* Taiwan, *THA* Thailand.

**Figure 2:**
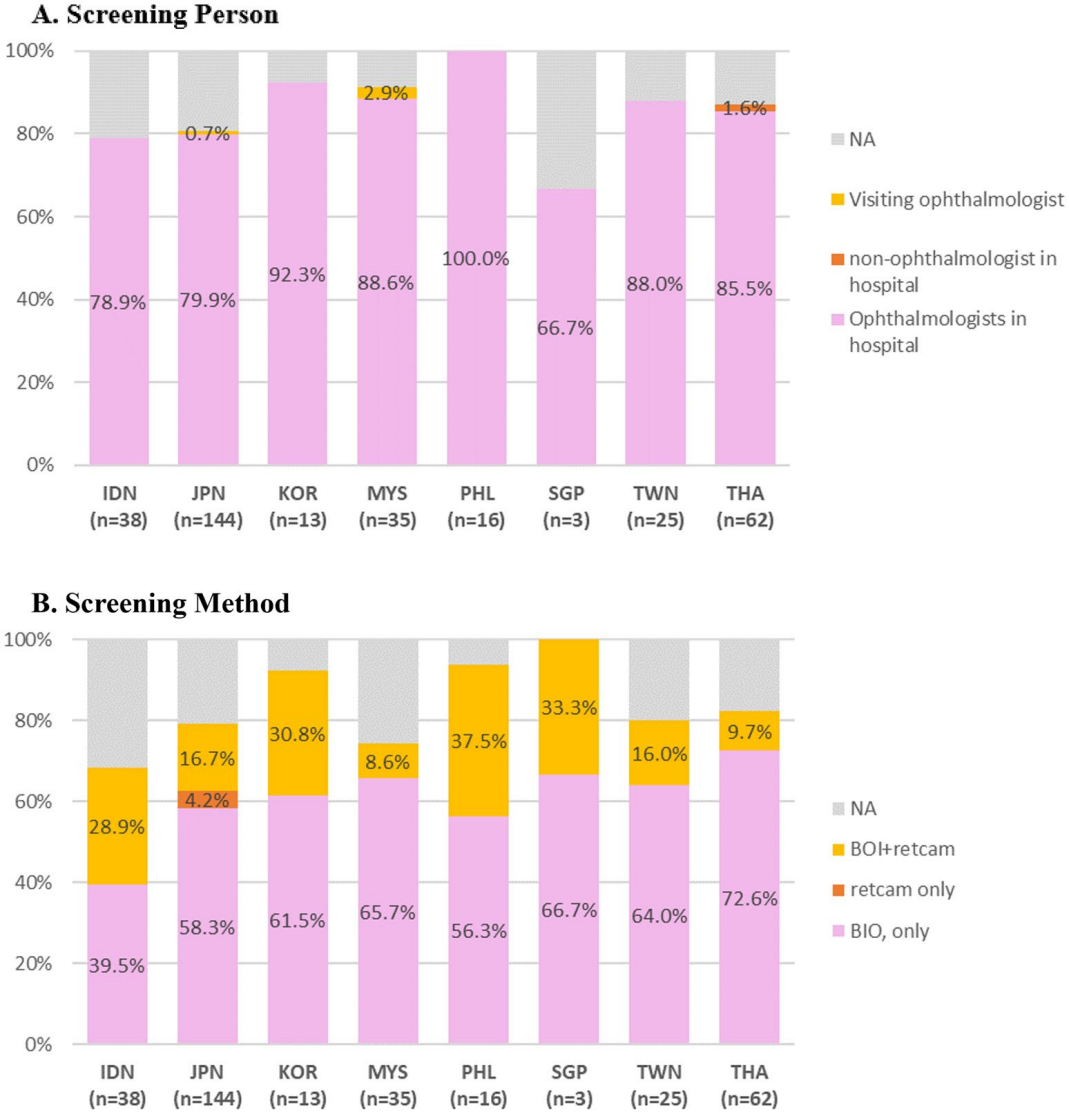
The way of initial eye examination for ROP screening in the eight AsianNeo Countries. (**A**) Screening person. (**B**) Screening method. *BIO* bilateral indirect ophthalmoscopy, *IDN* Indonesia, *JPN* Japan, *KOR* South Korea, *MYS* Malaysia, *NA* not available, *PHL* Philippines, *SGP* Singapore, *TWN* Taiwan, *THA* Thailand.

A majority of NICUs used screening criteria based on a either GA and/or birth weight cut-off. One NICU in Indonesia did not have GA based criteria, and several NICUs did not have birth weight-based criteria: six in Indonesia, 43 in Japan, 4 in The Philippines, 3 in Taiwan, and 6 in Thailand. In Indonesia and Japan, there were many NICUs in which ROP screening was performed in infants born at less than 34 weeks. In South Korea, Malaysia, and Taiwan, NICUs screening for ROP in infants born at less than 32 weeks of GA were most common. Although there were various weight criteria from less than 1000 g to less than 2000 g, in all networks, highest number of NICUs answered if the infants weigh less than 1500 g, they conducted ROP screening exam. It is to note that many Asian countries have ROP screening criteria for less than 34 weeks of GA. Japan especially showed that the majority of NICU units screened ROP exam for later preterm babies. In Indonesia, infants who needed to be screened were transferred to other hospital for examination. In most NICUs, in-hospital ophthalmologists performed BIO, only in one NICU from Thailand, the initial examination was undertaken by a non-ophthalmologist. At the national level, BIO and wide-field digital ROP screening method were used together, but at the hospital (unit) level, there were places where only BIO was possible. And digital ROP screening method was used as an auxiliary method for BIO, and six NICUs in Japan that used tele-digital imaging of retina as a single initial screening test (Figs. 1 and 2).

### Treatment

**Figure 3:**
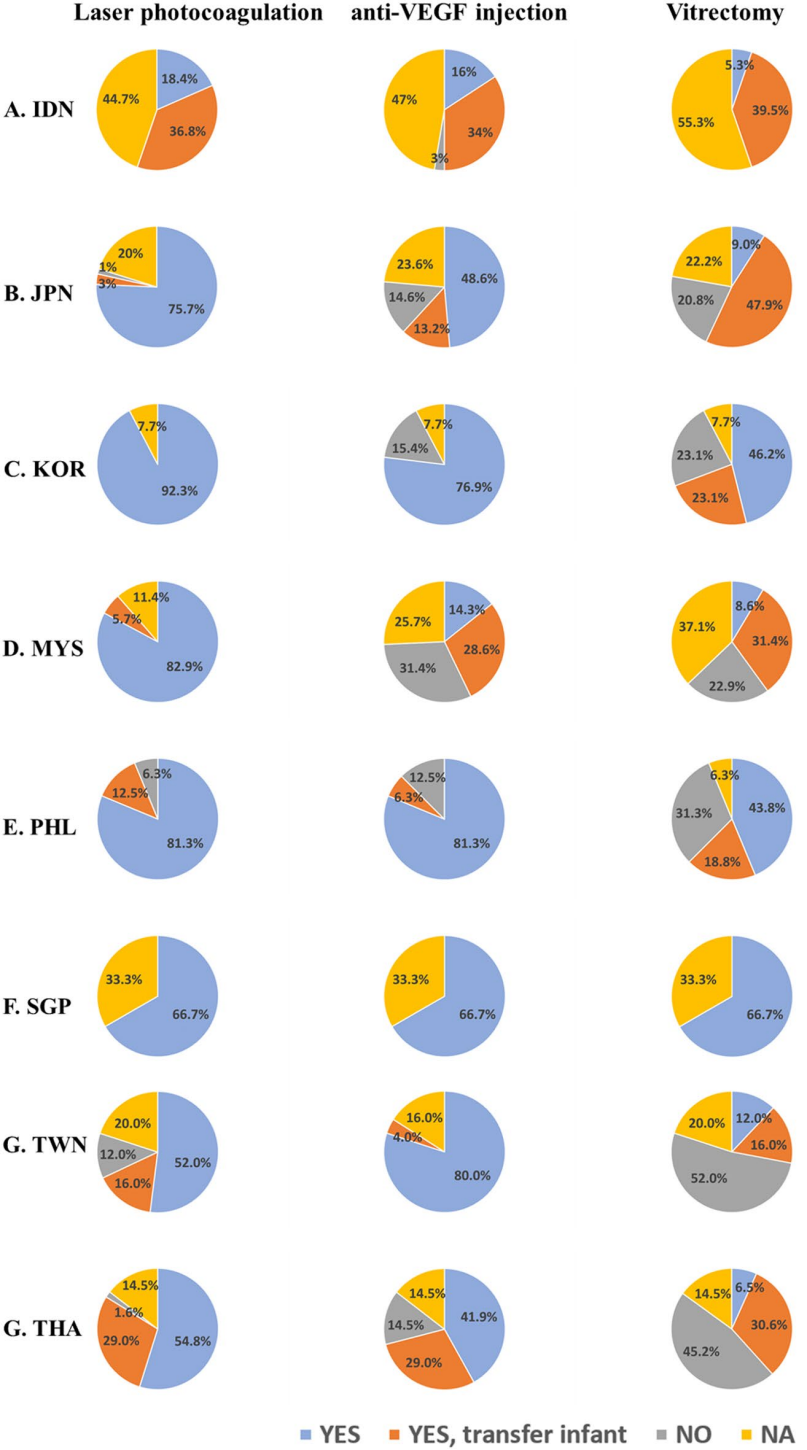
Treatment strategies of ROP in the eight AsianNeo Countries. The left column is about the laser photocoagulation, the middle is about the anti-VEGF injection, and the right column is the result of the vitrectomy. (**A**) Indonesia (n = 38); (**B**) Japan (n = 144); (**C**) South Korea (n = 13); (**D**) Malaysia (n = 35); (**E**) Philippines (n = 16); F, Singapore (n = 3); (**G**) Taipei (n = 25); (**H**) Thailand (n = 62). *IDN* Indonesia, *JPN* Japan, *KOR* South Korea, *MYS* Malaysia, *NA* not available, *PHL* Philippines, *SGP* Singapore, *TWN* Taiwan, *THA* Thailand, *VEGF* vascular endothelial growth factor;

Like worldwide, the current standard for ROP treatment in the Asian region is laser photocoagulation delivered through laser indirect ophthalmoscopy, although there has been an increasing trend to use anti-vascular endothelial growth factor (anti-VEGF) agents more recently. Infants requiring treatment for ROP could be treated in-hospital or transferred to another hospital to receive laser therapy or retinal ablation: 21/38 units in Indonesia, 113/144 units in Japan, 12/13 units in South Korea, 31/35 units in Malaysia, 15/16 units in the Philippines, 2/3 units in Singapore, 17/25 units in Taiwan, and 52/62 units in Thailand. Another treatment method, anti-VEGF injection is also possible but there are less possible units than laser therapy: 19/38 units in Indonesia, 89/144 units in Japan, 10/13 units in South Korea, 15/35 units in Malaysia, 14/16 units in the Philippines, 2/3 units in Singapore, 21/25 units in Taiwan, and 44/62 units in Thailand. In the case of vitreous surgery, vitrectomy is conducted less frequently in all countries except for Singapore which was in 66.7% of the units surveyed (Fig. 3).

## Discussions

The current national ROP guidelines use the GA and birth weight for screening. Most western guidelines focus on infants born with birth weight of less than 1500 g, or GA of less than 31 weeks. In the US, the AAP and American Academy of Ophthalmology recommended infants ≤ 30^+6^ weeks of GA or ≤ 1500 g of birth weight to be screened for ROP^24^. Although the current Canadian screening guidelines included infants of ≤ 31^+6^ weeks of GA or ≤ 1250 g of birth weight, the risk of ROP was reported to be the highest in the screened infants ≤ 27^+6^ weeks GA and/or ≤ 750 g birth weight (62.9%), which was the most restrictive threshold of all studies^33^. However, the criteria for ROP screening guidelines around the Asian countries range higher than the recommended guidelines of US and UK ranging from 30 to 37 weeks of GA and BW from < 1000 to 2500 g. In our observation of the 8 Asian countries, Japan mostly showed ROP screening guide for < 34 weeks of GA. There was a trend to screen ROP in later preterm babies > 30 weeks of GA in other countries as well. Overall, the ROP screening criteria of GA and birth weight among Asian countries varies widely. The oxygen target range is similar between the countries in our survey. The lower limit was 88% and the highest limit was 95%. The UK’s National Institute for Health and Care Excellence (NICE) guidelines recommend a target oxygen saturation of 91–95% in preterm infants born at less than 32 weeks of gestation^25^ and an AAP clinical report also favored an oxygen saturation target of approximately 90–95%^34^.

ROP screening guidelines may vary over time in some countries depending on their economic development, population composition and medical level circumstances: American Ophthalmological Society updated the ROP screening guidelines in 2006^35^, 2013^36^, and 2018^24^. However, an approach to establish more targeted screening criteria for ROP may minimize unnecessary number of infants going through ROP screening procedure and reduce the healthcare provider’s work burden. Because the risk for severe ROP and vision loss will vary from country to country, especially from high to middle and low income countries, significant research efforts to develop innovative evidence-based ROP screening algorithms with greater specificity can be also adopted in Asian countries^37^. Further analysis of the association between economic development and availability of different treatment modalities would be an interesting topic to pursue. These efforts will eventually lead to more structured ROP screening and follow-up guidelines ensuring those at high-risk for needing treatment are not missed at the same time.

As an on-line survey, detailed information of individual participating unit (countries or center) could not be collected. As shown in Table 1, some had a low ROP incidence of 6.7%. This low rate appears significantly lower than most Western countries: For instance, the USA has an any ROP rate of 70.6% among infants under 28 weeks of gestation^20^, the UK has a rate of 12.6% among VLBW infants^23^. Possible explanation for this low incidence might be as follows: (1) extreme preterm infants such as 24–26 weeks of gestation who were at high risk of ROP development were not included in the denominator due to low survival rate, (2) ROP screening was missed in more infants than expected, and (3) preterm infants that require ROP screening exam were transferred to other hospital. Each of these possibilities seems to be an important issue that can be addressed in the future to improve quality of NICU care. The survey was replied by neonatologists working in general or universal hospitals and they may have compliant ROP screening programs compared to lower-level hospitals in each country.

Through a large international survey from 10 networks and 11 countries contributing to iNeo, and a survey of 200 European NICUs, most of NICUs had changed their SpO_2_ limits in the neonatal practice over a decade with new limits based on strong scientific evidence in support of these changes^38,39^. In the future, the Asian neonatal networks and research trials may also enable to collaborate to facilitate quality improvement (QI) by adopting better practices and further enhance systematic support through national welfare policies. As an example, QI programs facilitated by the California Perinatal Quality Care Collaborative identified ways to improve ROP screening in the NICU and reduced severe ROP with improved delivery room management^40^. With rapid technical advances in machine learning, it has permeated into nearly every sector of science including ophthalmology. Retinal imaging through convolutional neural network can be also applied to image-based pattern recognition which is suited for diagnosis and may be integrated to further management. The diagnosis of ROP can be more easily detected with better prediction of intervention: laser photocoagulation or anti-VEGF injection. Overall, the integration of machine learning into ophthalmology holds great promise in enhancing diagnostic capabilities and improving patient outcomes in the field of retinal imaging. The use of advances in technology may also enable us to provide universal strategy for preventable blindness from ROP coupled with timely diagnosis and treatment in the near future.

There are several strengths in this project. Firstly, it is the first paper to compare ROP practices implemented in eight Asian countries. The leading neonatologists of each country or region participate in this project as steering committee members. Even though the participating units are small to represent the national policy, the leading participating units mostly reflect their national policy. For example, the 13 KNN executive member units out of a total 76 units (17%) from Korea represents the nation-wise policy and has little variation in ROP screening. Secondly, compared with the other international collaboration such as the iNEO which involves countries across the globe, this project has a geographically close collaboration representing Asian neonates. Thirdly, wide variations in the NICU systems, resource availabilities, ethnicities, and cultural backgrounds among participating countries or regions will provide unique opportunities to assess how these variations affect their clinical practices in NICUs and the following outcomes. This will further lead to expanding quality improvement throughout the collaboration across Asia.

However, this study has a few limitations. Firstly, even though the leading neonatologists of each country participated in the survey, the policy may not always represent the national guidelines and only reflect the practices of individual NICUs. The proportion of the participation varies among the countries or regions and the survey questions can be answered reflecting only regional part of the area. Unfortunately, the AsianNeo registry cannot currently reflect a unified screening criteria that represents each country. Second, the retrospective nature of study to describe and compare the outcomes of VLBW infants among Asian countries and regions may be prone to recall or misclassification bias. Third, this was a study based on an on-line survey, further information on some responses was not possible concerning the survey results. For example, specific reasons for the high rate of vitrectomy remains unclear from the survey. Lastly, as a part of multi-national survey, more detailed information in relation to ROP was not obtained to make any significant associations.

In conclusion, the burden of the ROP is enormous, with a variety of screening standards; a significant part of Asia is still suffering from the third epidemic of ROP. Systematic approach should be initiated from the time of birth to prevent and screen for ROP as needed per clinical environment rather than just following the guidelines of another country. The incidence of ROP may be a comprehensive marker for the quality of NICU care as it encompasses many tiers of neonatal care. QI projects may be needed to find the root cause if an increased incidence of ROP is noted. Combined efforts and continuous QI work are necessary not only to educate but also to retrospectively review the practices concerning ROP. The AsianNeo survey project enabled us to evaluate the information regarding perinatal care systems, clinical practice and outcomes of participating countries or regions. The information will serve as a foundation for future QI activities of perinatal care in participating countries. For the educational and QI projects in AsianNeo countries and regions, the development of an AsianNeo registry with uniform variables and maintaining a nation-wide database is important. Through these projects, the AsianNeo will provide an international platform for pediatricians or neonatologists, researchers, other health care providers to understand the health system, clinical management and outcomes in Asian countries or regions to learn from each other and improve the quality of neonatal care. AsainNeo is speculated to help member NICUs in overcoming this ROP epidemic.

## Methods

### Structure of the AsianNeo

AsianNeo is an international collaboration which is composed of national or regional, population-based neonatal networks and/or registries including 8 networks from 8 Asian countries: the network from Indonesian pediatric society, NRNJ from Japan, KNN from South Korea, MNNR from Malaysia, the network from Philippine Society of Newborn Medicine, the network from level III–IV public hospitals in Singapore, TNN from Taiwan, and the network from Thai Neonatal Society. There is no national registry of VLBW infants in Singapore, however, 3 NICUs manage more than 80% of VLBW infants born in the country and each of them have their own unit database of VLBW infants. The AsianNeo aims to provide an international platform to assess and understand the health systems, the clinical management, and the outcomes of small or sick newborn infants in Asia. Through this, the quality of neonatal care and the outcomes of the infants in the Asian countries or regions as well as global could be improved. The neonatal networks and working members of the AsianNeo got together at monthly on-line and a yearly face-to-face meeting and they collaborate through surveys, clinical research, and quality improvement activities.

### On-line survey

We have conducted a cross-sectional, international, on-line survey at the institutional level to assess the human material resources and clinical practice protocol of very preterm infants. A questionnaire on treatment practices relating to the VLBW (birth weight < 1500 g) or very preterm infants (birth at < 32 weeks gestation) were sent to the neonatologist directors of 336 tertiary NICUs in 8 collaborating population-based networks reflecting their unit practice/protocols, based on their 2021 standards, and not personal preferences: Indonesia (n = 38), Japan (n = 144), South Korea (n = 13), Malaysia (n = 35), Philippines (n = 16), Singapore (n = 3), Taiwan (n = 25) and Thailand (n = 62). Differing care practices and treatment criteria were compared among countries (Table 1). The questionnaire was written in English and coded into the Survey Monkey® online tool, and answers were collected in English or translated into native languages for non-English-speaking countries. We confirm that all methods were performed in accordance with the relevant guidelines and regulations of AsianNeo Bureau.

### Data analysis

Data are reported using descriptive statistics. For ROP screening criteria, stacked bar graphs for gestational age (GA) and birth weight was expressed for plotting distribution. Additionally, we reviewed previously published studies, and investigated the incidence of ROP and screening guidelines by countries.

### Ethics approval

This study protocol has been approved by the research ethics board of the National Center for Child Health and Development (NCCHD), Tokyo, Japan (ID 2020-244) and responsible for the research. From the survey, system, clinical management protocols, and outcomes for QI of each NICUs were collected, and any individual patient data was not involved in the survey therefore, we obtained written informed consents from the neonatologist who answered the on-line survey. All the networks will sign data transfer agreements with the NCCHD before the data transfer. Informed consent was exempted because no data identifiable at a patient level will be collected or transmitted to the AsianNeo bureau, and only aggregate data will be reported in public.

## Data Availability

The dataset analyzed in this study is not publicly available due to the policy of AsianNeo. However, datasets are available from the corresponding author upon reasonable request.

## References

[1] Terry T (1943). Fibroblastic overgrowth of persistent tunica vasculosa lentis in premature infants: II. Report of cases—Clinical aspects. Arch. Ophthalmol..

[2] Blencowe H, Lawn JE, Vazquez T, Fielder A, Gilbert C (2013). Preterm-associated visual impairment and estimates of retinopathy of prematurity at regional and global levels for 2010. Pediatr. Res..

[3] Perin J (2022). Global, regional, and national causes of under-5 mortality in 2000–19: An updated systematic analysis with implications for the sustainable development goals. Lancet Child Adolesc. Health.

[4] Cross K (1973). Cost of preventing retrolental fibroplasia?. Lancet.

[5] Blencowe H (2013). Born too soon: The global epidemiology of 15 million preterm births. Reprod. Health.

[6] Lucey JF, Dangman B (1984). A reexamination of the role of oxygen in retrolental fibroplasia. Pediatrics.

[7] Gilbert C (2008). Retinopathy of prematurity: A global perspective of the epidemics, population of babies at risk and implications for control. Early Hum. Dev..

[8] Chang YS, Park H-Y, Park WS (2015). The Korean neonatal network: An overview. J. Korean Med. Sci..

[9] Investigators of the Vermont-Oxford Trials Network Database Project (1993). The Vermont-Oxford trials network: Very low birth weight outcomes for 1990. Pediatrics.

[10] Shah PS (2019). The international network for evaluating outcomes (iNeo) of neonates: Evolution, progress and opportunities. Transl. Pediatr..

[11] Chawanpaiboon S (2019). Global, regional, and national estimates of levels of preterm birth in 2014: A systematic review and modelling analysis. Lancet Glob. Health.

[12] Siswanto JE (2021). Multicentre survey of retinopathy of prematurity in Indonesia. BMJ Paediatr. Open.

[13] Kono Y, Neonatal Research Network of Japan (2021). Neurodevelopmental outcomes of very low birth weight infants in the Neonatal Research Network of Japan: Importance of neonatal intensive care unit graduate follow-up. Clin. Exp. Pediatr..

[14] Hwang JH, Lee EH, Kim EA-R (2015). Retinopathy of prematurity among very-low-birth-weight infants in Korea: Incidence, treatment, and risk factors. J. Korean Med. Sci..

[15] Choo MM (2021). Comparative cohorts of retinopathy of prematurity outcomes of differing oxygen saturation: Real-world outcomes. BMJ Open Ophthalmol..

[16] Paulino JAT, Santiago APD, Santiago DE (2020). Comparison of the detection rates for retinopathy of prematurity (ROP) of the 2013 Philippine Academy of Ophthalmology (PAO) Revised Philippine guidelines and the 2005 PAO-Philippine Pediatric Society (PPS) guidelines for ROP screening in the Philippine General Hospital: a 5-year review. BMJ Open Ophthalmol..

[17] Lee J (2023). Trends in neonatal mortality and morbidity in very-low-birth-weight (VLBW) infants over a decade: Singapore national cohort study. Pediatr. Neonatol..

[18] Kang EY-C (2018). Retinopathy of prematurity trends in Taiwan: A 10-year nationwide population study. Investig. Ophthalmol. Vis. Sci..

[19] Ittarat M, Chansaengpetch S, Chansangpetch S (2023). Incidence and risk factors for retinopathy of prematurity at a rural Tertiary Hospital in Thailand. J. Ophthalmic Vis. Res. (JOVR).

[20] Natarajan G (2019). Neurodevelopmental outcomes of preterm infants with retinopathy of prematurity by treatment. Pediatrics.

[21] Ludwig CA, Chen TA, Hernandez-Boussard T, Moshfeghi AA, Moshfeghi DM (2017). The epidemiology of retinopathy of prematurity in the United States. Ophthalmic Surg. Lasers Imaging Retina.

[22] Adams GG (2017). Treatment trends for retinopathy of prematurity in the UK: Active surveillance study of infants at risk. BMJ Open.

[23] Painter SL, Wilkinson AR, Desai P, Goldacre MJ, Patel C (2015). Incidence and treatment of retinopathy of prematurity in England between 1990 and 2011: Database study. Br. J. Ophthalmol..

[24] Fierson WM (2018). Screening examination of premature infants for retinopathy of prematurity. Pediatrics.

[25] Wilkinson A (2023). UK screening and treatment of retinopathy of prematurity updated 2022 guidelines. Early Hum. Dev..

[26] Siswanto JE (2021). How to prevent ROP in preterm infants in Indonesia?. Health Sci. Rep..

[27] Philippine Pediatric Society, Philippine Academy of Ophthalmology, Retinopathy of Prematurity Working Group (2020). Retinopathy of prematurity Philippine preventive care plan strategy. Philipp. J. Ophthalmol..

[28] Shah V, Yeo C, Ling Y, Ho L (2005). Incidence, risk factors of retinopathy of prematurity among very low birth weight infants in Singapore. Ann. Acad. Med. Singap..

[29] Malaysia, M. H. M. A. M. *Clinical Practice Guidelines. Retinopathy of Prematurity*. https://www.moh.gov.my/moh/attachments/3917.pdf (2005).

[30] Chen Y-H (2015). Natural history of retinopathy of prematurity: Two-year outcomes of a prospective study. Retina.

[31] Ikeda N (2001). Retinopathy of prematurity in Toyama area of Japan. Ann. Ophthalmol..

[32] Choi SY (2014). Retinopathy of prematurity in infants with birth weights greater than 1,000 grams. Neonatal Med..

[33] Borroni C (2013). Survey on retinopathy of prematurity (ROP) in Italy. Ital. J. Pediatr..

[34] Tarnow-Mordi W, Kirby A (2019). Current recommendations and practice of oxygen therapy in preterm infants. Clin. Perinatol..

[35] Lichtenstein SJ (2006). Screening examination of premature infants for retinopathy of prematurity. Pediatrics.

[36] American Academy of Pediatrics Section on Ophthalmology (2013). Screening examination of premature infants for retinopathy of prematurity. Pediatrics.

[37] Wade KC (2015). Safety of retinopathy of prematurity examination and imaging in premature infants. J. Pediatr..

[38] Huizing MJ, Villamor-Martínez E, Vento M, Villamor E (2017). Pulse oximeter saturation target limits for preterm infants: A survey among European neonatal intensive care units. Eur. J. Pediatr..

[39] Darlow BA (2018). Variations in oxygen saturation targeting, and retinopathy of prematurity screening and treatment criteria in neonatal intensive care units: An international survey. Neonatology.

[40] Lapcharoensap W (2017). Effects of delivery room quality improvement on premature infant outcomes. J. Perinatol..

